# Targeting the cancer stem cell marker, aldehyde dehydrogenase 1, to circumvent cisplatin resistance in NSCLC

**DOI:** 10.18632/oncotarget.19881

**Published:** 2017-08-03

**Authors:** Lauren MacDonagh, Michael F. Gallagher, Brendan Ffrench, Claudia Gasch, Eamon Breen, Steven G. Gray, Siobhan Nicholson, Niamh Leonard, Ronan Ryan, Vincent Young, John J. O'Leary, Sinead Cuffe, Stephen P. Finn, Kenneth J. O'Byrne, Martin P. Barr

**Affiliations:** ^1^ Thoracic Oncology Research Group, School of Clinical Medicine, Trinity Translational Medicine Institute, Trinity Centre for Health Sciences, St. James's Hospital & Trinity College, Dublin, Ireland; ^2^ Histopathology Department, Trinity College Dublin, Sir Patrick Dun Laboratories & Central Pathology Laboratory, St. James's Hospital, Pathology Research Laboratory, Coombe Women and Infant's University Hospital, Dublin, Ireland; ^3^ Flow Cytometry Facility, Trinity Translational Medicine Institute, Trinity Centre for Health Sciences, St. James's Hospital & Trinity College, Dublin, Ireland; ^4^ Histopathology Department, St. James's Hospital, Dublin, Ireland; ^5^ Department of Cardiothoracic Surgery, St. James's Hospital, Dublin, Ireland; ^6^ Cancer & Ageing Research Program, Queensland University of Technology, Brisbane, Australia

**Keywords:** cancer stem cell, cisplatin, resistance, ALDH1, NSCLC

## Abstract

Non-small cell lung cancer (NSCLC) accounts for a large proportion of cancer deaths and is characterized by low treatment response rates and poor overall prognosis. In the absence of specific treatable mutations, cisplatin-based chemotherapy plays an important role in the treatment of this disease. Unfortunately, the development of resistance has become a major therapeutic challenge in the use of this cytotoxic drug. Elucidating the mechanisms underlying this resistance phenotype, may result in the development of novel agents that enhance sensitivity to cisplatin in lung cancer patients. In this study, targeting the cancer stem cell activity of aldehyde dehydrogenase 1 (ALDH1) was investigated as a strategy to overcome chemoresistance in NSCLC. Tumors from NSCLC patients showed an increase in their profile of pluripotent stemness genes. Cisplatin exposure induced the emergence or expansion of an ALDH1-positive subpopulation in cisplatin sensitive and resistant NSCLC cell lines, respectively, further enhancing cisplatin resistance. Using the Aldefluor assay and FACS analysis, ALDH1 subpopulations were isolated and evaluated in terms of stem cell characteristics. Only ALDH1-positive cells exhibited asymmetric division, cisplatin resistance and increased expression of stem cell factors *in vitro*. Xenograft studies in NOD/SCID mice demonstrated efficient tumorigenesis from low cell numbers of ALDH1-positive and ALDH1-negative subpopulations. Targeting ALDH1 with Diethylaminobenzaldehyde (DEAB) and Disulfiram, significantly re-sensitized resistant lung cancer cells to the cytotoxic effects of cisplatin. Our data demonstrate the existence of a lung CSC population and suggest a role for targeting ALDH1 as a potential therapeutic strategy in re-sensitizing NSCLC cells to the cytotoxic effects of cisplatin.

## INTRODUCTION

With more than one million cases of lung cancer diagnosed each year, it has become the leading cause of cancer-related death worldwide. Lung cancer is classified into two main subtypes; non-small cell lung cancer (NSCLC) which accounts for 85% of cases, while the remaining 15% consists of small-cell lung cancer (SCLC) [1, 2]. NSCLC is further divided into three sub-classifications; adenocarcinoma, squamous cell carcinoma and to a lesser extent, large cell carcinoma [3]. While NSCLC shows an initial response to chemotherapy, 5-year survival rates remains low at approximately 16%. This is largely due to the emergence of resistance prior to, and during treatment with chemotherapy and radiation therapy and as such, poses a significant challenge in the clinical setting [4].

Since its introduction into clinical trials in 1971 and subsequent FDA-approval in 1978, cis-diammine-dichloro-platinum II (cisplatin) represents a major landmark in the history of successful anti-cancer therapeutics [5]. It has changed the management of several solid malignancies, including lung cancer. Unfortunately, the development of cisplatin resistance has become a major clinical challenge in the treatment and management of lung cancer patients. Alternative strategies to overcome cisplatin resistance are of critical importance in order to enhance the current therapeutic efficacy of this chemotherapeutic drug.

The cancer stem cell (CSC) hypothesis suggests that a rare population of cells exist within tumors which display stem-like characteristics such as multipotency, increased expression of stemness-associated markers and the ability to self-renew and differentiate, both of which are essential for tumor initiation, maintenance, progression and metastasis [6, 7]. Cellular heterogeneity is a histological hallmark of many solid tumors. As such, the CSC hypothesis would suggest that the heterogeneity observed in phenotypically diverse tumors may arise due to the hierarchical cell dynamics produced as a result of asymmetric division and differentiation of this CSC population [8–9]. The ability of CSCs to asymmetrically divide enables these cells to simultaneously self-perpetuate and to generate differentiated progeny, thus giving rise to a heterogeneous tumor with a consistently maintained CSC population [10–12]. In addition, it has previously been shown that the CSC population expands during periods of stress, such as that induced by exposure to chemotherapeutic agents. The existence of lung CSCs may explain why tumors exhibit resistance to a broad spectrum of chemotherapeutic agents that target and kill the bulk of the tumor and induce expansion and enrichment of the CSC subset [13–18].

Members of the aldehyde dehydrogenase (ALDH) family of cytosolic isoenzymes are responsible for oxidising intracellular aldehydes and play a role in the oxidation of retinol to retinoic acid in early stem cell differentiation [19]. Haematopoietic and neural stem cells display high ALDH activity [20, 21]. Increased ALDH1 activity has been observed in stem cell populations and more recently, ALDH1 has been identified as a promising CSC marker in a number of malignancies, including lung cancer [22–26]. While ALDH1 activity has been reported in a number of NSCLC cell lines and tumor samples, it's role in chemotherapeutic resistance has not, as yet, been fully elucidated [27, 28]. In this study, we examined the expression and function of ALDH1 as a stemness marker. Furthermore, the effects of targeting ALDH1 by chemical and pharmacological inhibition were assessed in terms of their ability to re-sensitize resistant lung tumor cells to the cytotoxic effects of cisplatin.

## RESULTS

### NSCLC tumors show increased expression of a distinct stemness gene profile

**Figure 1:**
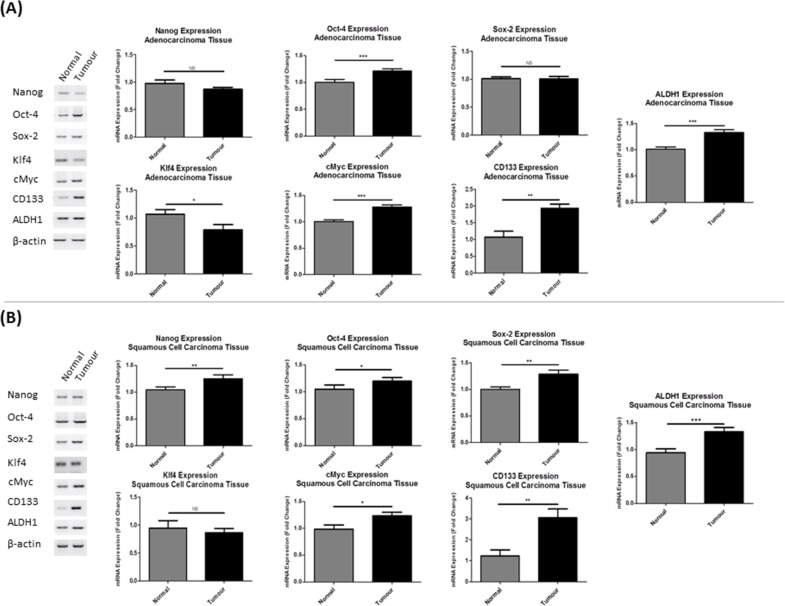
Lung tumor tissues show differential expression of pluripotent stemness genes Gene expression analysis of stemness genes and CSC markers were assessed in **(A)** adenocarcinoma and **(B)** squamous cell carcinoma tissues from NSCLC patients (n=20) relative to matched normal lung tissues by RT-PCR. *NANOG*, *OCT-4*, *SOX-2*, *KLF4*, *C-MYC*, *CD133* and *ALDH1* were significantly altered in both tumor subtypes. Data are shown for adenocarcinoma (n=10) and squamous cell carcinoma (n=10) patient tumor and matched normal lung tissue samples and are represented as Mean ± SEM (*p<0.05, **p<0.01, ***p<0.001).

RT-PCR and densitometric quantification analysis was used to quantify mRNA expression of a panel of stemness genes (*NANOG*, *OCT-4*, *SOX-2*, *KLF4* and *C-MYC*), in addition to the CSC-specific markers*, CD133* and *ALDH1*, in a cohort of matched normal and tumor lung tissues from NSCLC patients (n=20). Expression of the stemness-associated gene profile was significantly altered in both adenocarcinoma (Figure 1A) and squamous cell carcinoma tumor tissues (Figure 1B) relative to their matched normal lung tissues. Within the cohort of adenocarcinoma patients, there was a significant change in expression of the Yamanaka factors, *OCT-4* (p<0.001), *KLF4* (p<0.05) and *C-MYC* (p<0.001). Similarly, gene expression of the CSC markers, *CD133* (p<0.01) and *ALDH1* (p<0.001) were significantly altered in tumor tissues. A similar, but more significant increase in the number of cancer stemness genes was also found in squamous cell carcinoma patients, *NANOG* (p<0.01), *OCT-4* (p<0.05), *SOX-2* (p<0.01), *C-MYC* (p<0.05). *CD133* (p<0.01) and *ALDH1* (p<0.001) mRNA was significantly up-regulated in squamous cell tumors. These data imply a greater stem-like population in NSCLC tumors relative to their matched normal tissues.

### Cisplatin resistant NSCLC cells exhibit enhanced ALDH1 activity

**Figure 2:**
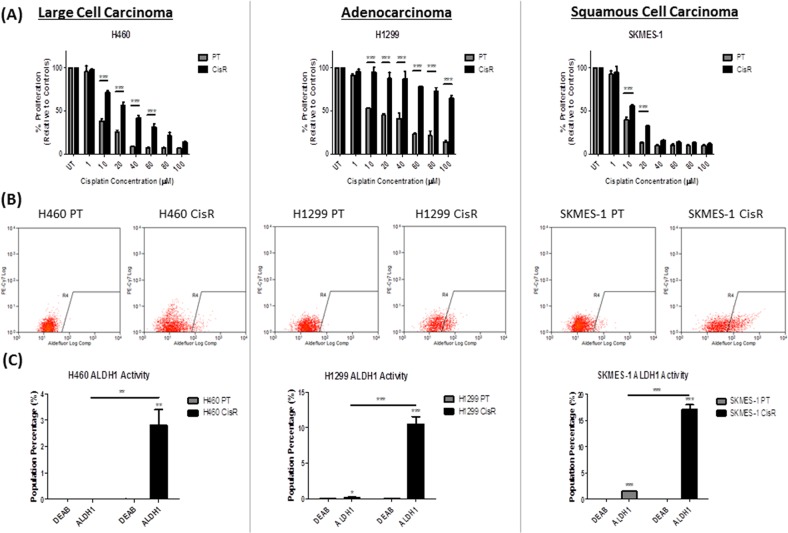
ALDH1 activity is increased in cisplatin resistant NSCLC cells Parental (PT) and cisplatin resistant (CisR) NSCLC cell lines were treated with increasing concentrations of cisplatin (0-100μM) for 72hrs. **(A)** Proliferation was measured by BrdU where cisplatin resistant sublines showed a significantly greater proliferative capacity when challenged with cisplatin relative to their parental counterparts. **(B)** ALDH1 activity was measured by flow cytometry using the Aldefluor assay. ALDH1 activity was determined relative to negative controls for each cell line. The ALDH1 specific inhibitor DEAB was used to determine background fluorescence and thereafter, from which gates were set. **(C)** Cisplatin resistant cells showed significantly greater ALDH1 activity as measured by the increase in ALDH1+ve cells relative to their internal DEAB controls and parental cells **(C)**. Data are shown for three independent experiments and are represented as Mean ± SEM (*p<0.05, **p<0.01, ***p<0.001).

A panel of isogenic cisplatin resistant NSCLC cell lines were previously established in our laboratory [29]. Cisplatin resistant sublines (CisR) and their parental counterparts (PT) were treated with increasing concentrations of cisplatin (0-100μM) for 72hrs. H460, H1299 and SKMES-1 CisR sublines showed significantly greater resistance to cisplatin at varying concentrations, relative to their corresponding PT cells (Figure 2A).

The Aldefluor assay was used to investigate ALDH1 activity within the NSCLC panel of PT and CisR cell lines. Flow plots representing ALDH1 activity in H460, H1299 and SKMES-1 cell lines are shown (Figure 2B), where gating (R4) was defined for each cell line using cells treated with the ALDH1 inhibitor, DEAB. A significant increase in the presence of an ALDH1-positive (+ve) subpopulation was identified across all CisR sublines relative to their PT counterparts. The Aldefluor assay identified a distinct ALDH1+ve subpopulation, relative to DEAB controls in all cell lines with the exception of H460 PT cells (Figure 2C). Comparison of ALDH1 activity across PT and CisR sublines identified the presence of a significantly greater ALDH1+ve subpopulation in H460 (p<0.01), H1299 (p<0.001) and SKMES-1 (p<0.001) CisR sublines relative to their cisplatin sensitive counterparts. These data indicate that cisplatin resistant NSCLC cells are enriched for an ALDH1+ve cell subset.

### ALDH1-positive cells confer increased resistance to cisplatin and exhibit stem-like characteristics

**Figure 3:**
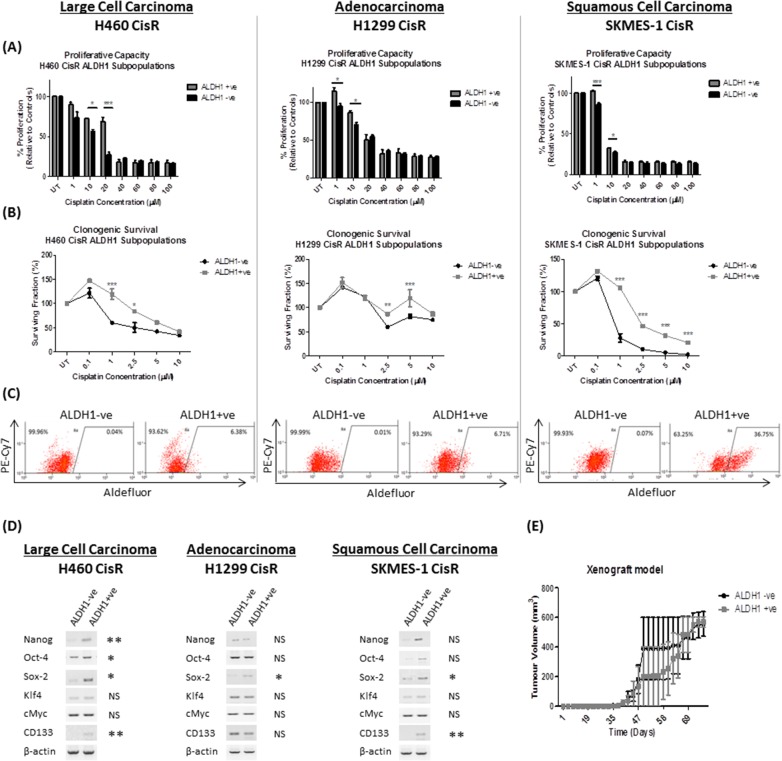
ALDH1-positive cells are resistant to cisplatin and exhibit distinct cancer stem cell properties Cisplatin resistant (CisR) sublines were stained using the Aldefluor assay and sorted into ALDH1+ve and ALDH1-ve subpopulations, with which a number of functional assays were performed to examine their stem-like characteristics. **(A)** ALDH1+ve and ALDH1-ve subpopulations were treated with increasing concentrations of cisplatin (0-100μM) for 72hrs and proliferation measured by BrdU. The ALDH1+ve cell subset showed a significantly greater proliferative capacity relative to their ALDH1-ve counterparts. **(B)** The clonogenic surviving fraction of ALDH1+ve cells was significantly greater when challenged with cisplatin compared to ALDH1-ve cells. **(C)** Asymmetric division assays were performed. A single ALDH1-ve cell gave rise to a progeny of ALDH1-ve cells in each chemoresistant cell line, while a single ALDH1+ve cell was capable of giving rise to a progeny of both ALDH1+ve and ALDH1-ve cells, confirming the ability of ALDH1+ve cells to asymmetrically divide. **(D)** Gene analysis (RT-PCR) of pluripotent stemness genes and CSC markers in ALDH1+ve and ALDH1-ve subpopulations showed significant differential expression of the stemness genes between ALDH1+ve and ALDH1-ve subpopulations. **(E)** To investigate the potential tumor forming ability of ALDH1 subpopulations, a xenograft mouse model was used for subcutaneous injection of ALDH1+ve and ALDH1-ve fractions isolated from the H460 CisR subline. While tumor growth was observed in NOD/SCID mice injected with ALDH1+ve and ALDH1-ve cell fractions, there was no significant difference in tumor initiation or growth between both cell subpopulations. Data are shown for three independent experiments and are represented as Mean ± SEM (*p<0.05, **p<0.01, ***p<0.001).

Cisplatin resistant sublines were stained using the Aldefluor assay and separated into ALDH1+ve and ALDH1-negative (−ve) cell fractions to examine the CSC potential of these subpopulations of cells. Cell fractions (ALDH1+ve and ALDH1-ve) were treated with increasing concentrations of cisplatin to assess their proliferative capacity (Figure 3A). The ALDH1+ve cell fractions isolated from each CisR cell line showed a significantly increased proliferative capacity in response to cisplatin, particularly at lower concentrations (1-10μM), relative to their ALDH1-ve controls. Similarly, the isolated ALDH1+ve fractions showed a significantly increased clonogenic survival ability at increasing concentrations of cisplatin (1-10μM) compared to the ALDH1-ve fractions across each of the NSCLC cell lines of different histological subtypes (Figure 3B).

Stem cells have the unique ability to asymmetrically divide, where a single stem-like cell has the potential to give rise to a heterogenic population. To examine the potential ability of ALDH1+ve cells to asymmetrically divide, single cells were isolated, plated and their ability to give rise to a heterogenic ALDH1 population was assessed by flow cytometry (Figure 3C). Clones arising from a single ALDH1-ve cell from H460, H1299 and SKMES-1 CisR sublines gave rise to a progeny consisting purely of ALDH1-ve cells only. However, cultures arising from a single ALDH1+ve cell from the same CisR sublines exhibited a mixed population of both ALDH1+ve and ALDH1-ve cells, indicating their ability to asymmetrically divide, thereby resulting in a differentiated progeny.

Furthermore, to determine whether ALDH1+ve cells expressed a distinct stem-like genotype, mRNA expression analysis of stemness and CSC-specific markers was investigated by RT-PCR (Figure 3D). While ALDH1+ve fractions from H460 CisR cells showed a significant increase in *NANOG* and *OCT-4* gene expression relative to their ALDH1-ve counterparts (2.75-fold, p<0.01 and 2.13-fold, p<0.05, respectively), *SOX-2* expression was significantly increased in ALDH1+ve fractions from all three CisR sublines (7.41-fold, p<0.05; 3.18-fold, p<0.05; 2.25-fold, p<0.05). Similarly, the CSC marker, *CD133*, was significantly up-regulated in H460 ALDH1+ve cells (11.12-fold, p<0.01) and SKMES-1 ALDH1+ve cells (2.25-fold, p<0.05) relative to their ALDH1-ve controls. To assess the tumor initiating capacity of ALDH1+ve subpopulations *in vivo*, ALDH1+ve and ALDH1-ve cells (1×10^3^) isolated from the H460 CisR subline, were subcutaneously injected into the right flank of NOD/SCID mice and tumor growth was measured over time (Figure 3E). ALDH1+ve and ALDH1-ve cell fractions efficiently gave rise to tumors.

While ALDH1+ve cells were more highly resistant to cisplatin, in contrast to their ALDH1-ve counterparts, they also exhibited the unique stem cell characteristic of asymmetric division resulting in unipotent lineage differentiation, in addition to increased expression of the embryonic and CSC panel of molecular markers relative to ALDH1-ve cells. Taken together, these data strongly suggest that ALDH1+ve cells are enriched for CSC characteristics and are present to a greater extent in cisplatin resistant NSCLC cells.

### Chronic exposure to cisplatin induces the emergence and expansion of an ALDH1-positive subpopulation

**Figure 4:**
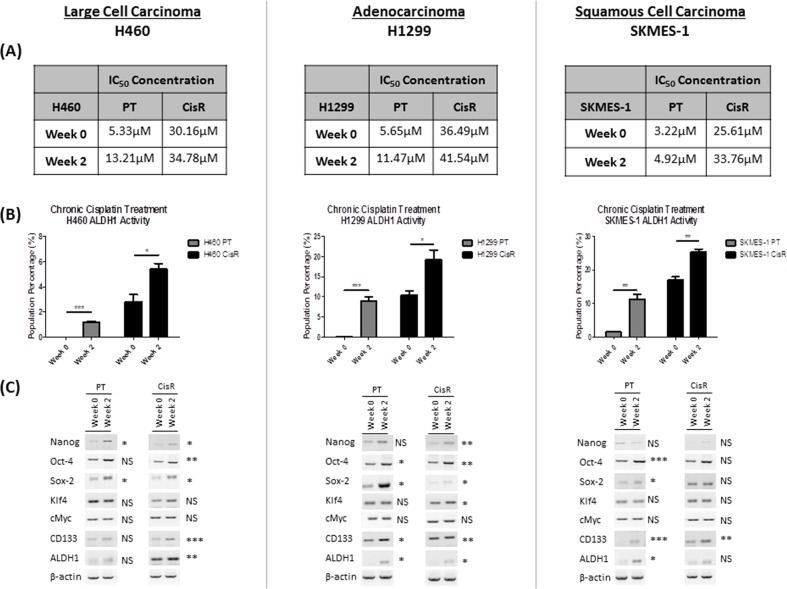
Chronic exposure to cisplatin selects for an ALDH1-positive cell subset PT and CisR sublines were chronically exposed for 2 weeks with 1μM and 10μM cisplatin, respectively. **(A)** Cells were subsequently treated with increasing concentrations of cisplatin (0-100μM) and proliferation was assessed by BrdU. Dose-response curves were used to deduce IC_50_ concentrations for cisplatin in each PT and CisR cell line. IC_50_ concentrations were increased following chronic exposure to cisplatin during this period compared to baseline controls at week 0. **(B)** Flow cytometry analysis of ALDH1 activity showed a significant expansion of this CSC population across all cell lines following prolonged exposure to cisplatin. **(C)** The effect of prolonged cisplatin exposure on stemness genes was examined by RT-PCR. A significant up-regulation of both stemness genes and CSC markers was observed at the mRNA level in PT and CisR cell lines relative to baseline controls (week 0). Data are shown for three independent experiments and are represented as Mean ± SEM (*p<0.05, **p<0.01, ***p<0.001).

To determine whether exposure to cisplatin may be responsible for the enrichment and expansion of the ALDH1+ve cell subset, PT and CisR cell lines were chronically exposed to 1μM and 10μM cisplatin, respectively, for 2 weeks to further promote resistance. After this time, cell lines were treated with increasing concentrations of cisplatin at baseline (week 0) and following chronic treatment (week 2) to determine the proliferative effects and resistance of this chemotherapeutic drug on these cells. Chronic exposure to cisplatin resulted in an increase in drug resistance, as demonstrated by differences in IC_50_ drug concentrations at baseline (week 0) and post-treatment (week 2) (Figure 4A).

The presence of the ALDH1+ve subpopulations were investigated following chronic exposure of the cell lines for two weeks, relative to controls (week 0) using the Aldefluor assay and flow cytometry analysis (Figure 4B). Chronic cisplatin exposure of the H460 PT cell line during this period, induced the emergence of a significant ALDH1+ve subpopulation (1.20±0.04%, p<0.001). At baseline, H460 PT cells have no detectable ALDH1 activity. This increase in ALDH1 at two weeks post-treatment, correlated with an increase in the IC_50_ concentration of cisplatin. Similarly, chronic exposure of the H460 CisR subline resulted in a significant expansion of the ALDH1+ve subset (2.81±0.59% vs 5.41±0.44%, p<0.05). This increase in ALDH1 was associated with a corresponding increase in the IC_50_ concentration of cisplatin, from 30.16μM (week 0) to 34.78μM (week 2). In H1299 cells, cisplatin exposure promoted the emergence of a highly significant ALDH1+ve CSC subset in PT cells (9.01±0.99%, p<0.001) and significantly enriched the ALDH1+ve subpopulation in the corresponding CisR subline from 10.5±1.02% to 19.17±2.43% (p<0.05). These observed increases in ALDH1+ve cell subsets were also associated with increased cisplatin IC_50_ concentrations and resistance to cisplatin (H1299 PT cells: 5.65μM vs 11.47μM; H1299 CisR cells: 36.49μM vs 41.54μM). Similar expansion of the ALDH1+ve subset was observed in SKMES-1 PT and CisR cells, where cisplatin significantly enhanced the presence of the ALDH1+ve subpopulations from 1.53±0.03% to 11.31±1.42% (p<0.01) in the SKMES-1 PT cell line and from 17.06±0.96% to 25.46±0.77% (p<0.01) in the SKMES-1 CisR subline. This in turn, corresponded with an increase in cisplatin IC_50_ concentrations (SKMES-1 PT cells: 3.22μM vs 4.92μM; SKMES-1 CisR cells: 25.61μM vs 33.76μM).

Based on our observations that ALDH1+ve cell subsets show increased expression of stemness markers relative to ALDH1-ve subsets, the stemness-associated mRNA gene profile of H460, H1299 and SKMES-1 cell lines following prolonged cisplatin exposure was examined. An up-regulation of both stemness and CSC-associated genes was observed (Figure 4C). Cisplatin significantly increased the expression of *NANOG* (p<0.05) and *SOX-2* (p<0.05) in the H460 PT cell line, while *NANOG* (p<0.05), *OCT-4* (p<0.01), *SOX-2* (p<0.05), in addition to the CSC-specific markers, *CD133* (p<0.001) and *ALDH1* (p<0.01), were significantly up-regulated in its CisR counterpart. In the H1299 PT cell line, cisplatin induced a significant induction of *OCT-4* (p<0.05), *SOX-2* (p<0.05), *CD133* (p<0.05) and *ALDH1* gene expression (p<0.05). In the CisR subline, there was a significant up-regulation of all markers, *NANOG* (p<0.01), *OCT-4* (p<0.01), *SOX-2* (p<0.05) and *KLF4* (p<0.05), *CD133* (p<0.01) and *ALDH1* (p<0.05), with the exception of *C-MYC*. While chronic treatment of the SKMES-1 PT cells with cisplatin significantly increased *OCT-4* (p<0.001), *SOX-2* (p<0.05), *CD133* (p<0.001) and *ALDH1* (p<0.05) mRNA, only *CD133* (p<0.01) was significantly altered in the the CisR subline relative to expression at baseline (week 0).

These data support our hypothesis that lung cancer cells exposed to cisplatin enrich for a subpopulation of ALDH1+ve cells with stem-like characteristics which may, at least in part, account for the resistance phenotype exhibited by these cells.

### ALDH1 inhibition reverses cisplatin resistance and re-sensitizes lung cancer cells to the cytotoxic effects of cisplatin

**Figure 5:**
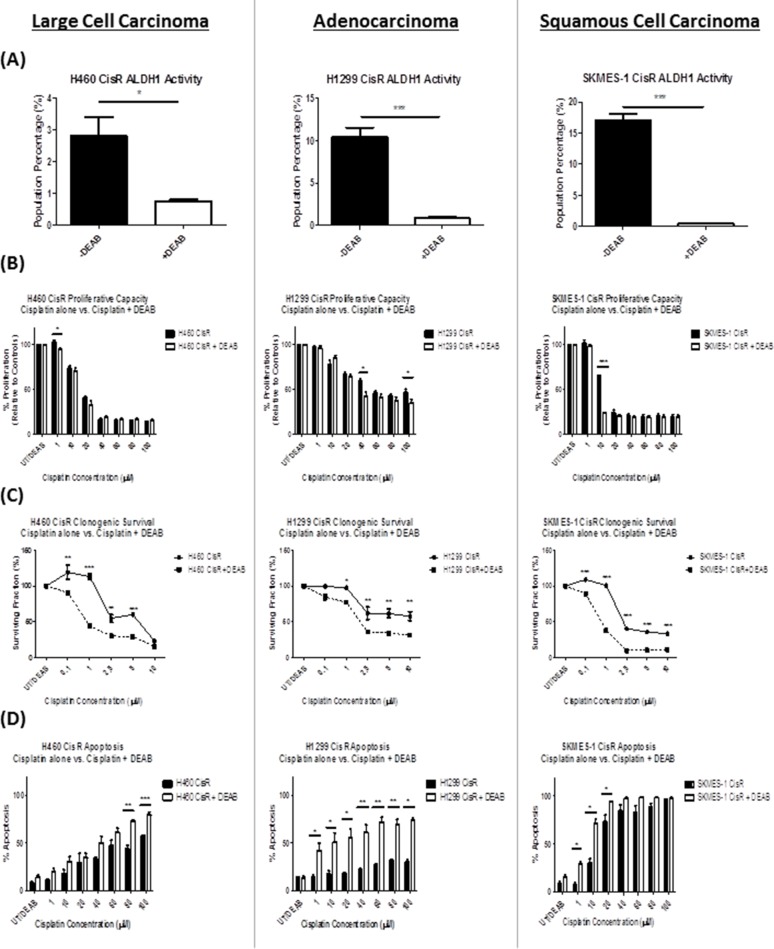
DEAB inhibition of ALDH1 reverses cisplatin resistance **(A)** Cisplatin resistant (CisR) sublines were treated with the ALDH1 inhibitor, DEAB (15μM) for 72hrs after which time, ALDH1+ve cell subpopulations were measured using the Aldefluor assay. DEAB significantly reduced the ALDH1+ve subpopulation across H460, H1299 and SKMES-1 CisR sublines. **(B)** The effects of DEAB alone, and in combination with increasing concentrations of cisplatin chemotherapy, on cell proliferation, were examined using the BrdU assay. DEAB significantly inhibited the proliferative capacity of CisR cells when used in combination with cisplatin relative to cisplatin only controls. **(C)** Similar effects were observed on the clonogenic survival of CisR cell lines where DEAB, in combination with cisplatin, significantly reduced the surviving fraction of NSCLC cells. **(D)** Cisplatin-induced cell death was assessed by flow cytometry using Annexin-V/PI staining. While DEAB alone did not induce apoptosis relative to untreated cells, DEAB in combination with cisplatin significantly increased apoptotic cell death across CisR cells relative to cisplatin only controls. Data are shown for three independent experiments and are represented as Mean ± SEM (*p<0.05, **p<0.01, ***p<0.001).

To assess whether inhibition of ALDH1 can overcome cisplatin resistance, CisR sublines were treated with the ALDH1 inhibitor, DEAB, for 72hrs and ALDH activity was reassessed by flow cytometry. A significant reduction in the ALDH1+ve cell subset was observed across all three cisplatin resistant sublines (Figure 5A) relative to untreated controls. In H460 CisR cells, the percentage of ALDH1+ve cells was significantly reduced from 2.81±0.59% to 0.76±0.05% (p<0.05), while in H1299 and SKMES-1 CisR cells, a similar reduction was observed in both cell lines, [H1299: 10.5±1.02% vs 0.9±0.07% (p<0.001); SKMES-1: 17.06±0.96% vs 0.48±0.01% (p<0.001)].

To further assess the functional effects of ALDH1 inhibition in cisplatin resistant NSCLC, cisplatin resistant sublines were treated with increasing concentrations of cisplatin (0-100μM) alone, and in combination with DEAB (15μM). The functional effects of inhibiting ALDH1 activity in combination with cisplatin treatment in CisR NSCLC sublines were examined in terms of proliferative capacity, clonogenic survival and apoptotic cell death relative to cisplatin-only treated cells (Figure 5). DEAB, in combination with cisplatin, significantly reduced proliferation across all three CisR sublines (Figure 5B) at varying concentrations of cisplatin. While this effect was evident across all three CisR sublines, albeit at different concentrations of cisplatin, DEAB significantly reduced the proliferation of H460, H1299 and SKMES-1 resistant cell lines at different concentrations of cisplatin (H460: 1μM; H1299: 40μM and 100μM; SKMES-1: 10μM). The clonogenic survival of cisplatin resistant sublines was significantly altered in response to treatment with DEAB when used in combination with cisplatin (Figure 5C). In all resistant sublines, there was a significant reduction in the number of surviving colonies at the different concentrations of cisplatin examined (0.1-10μM), when treated in combination with DEAB. These findings further support the targeting of ALDH1 in reversing resistance to cisplatin chemotherapy *in vitro*.

To ascertain the effect of DEAB on cisplatin-induced cell death, apoptosis was also measured. While DEAB alone did not induce apoptosis in the CisR sublines examined, relative to untreated cells, combination treatments did however induce a significant increase in apoptosis across all CisR sublines (Figure 5D). Of interest, this effect was most significant in the H1299 CisR subline, where DEAB in combination with cisplatin at concentrations ranging from 1-100μM, resulted in significant increases in apoptotic cell death in these cells compared to cisplatin-only treated cells. Collectively, treatment of cisplatin resistant sublines with DEAB significantly depleted an ALDH1+ve CSC subset, thereby sensitizing chemoresistant NSCLC cells to the cytotoxic effects of cisplatin.

### Therapeutic targeting of ALDH1 using Disulfiram reverses resistance of NSCLC cells to cisplatin

To examine the clinical potential of ALDH1 inhibition in cisplatin resistant lung cancer, CisR sublines were treated with low-doses (0.25μM) of Disulfiram, a potent FDA-approved ALDH inhibitor, together with equimolar copper chloride (CuCl_2_). The effect of Disulfiram has previously been shown to be potentiated by copper when used in combination *in vitro* and as such, all experiments carried out as part of this study employed Disulfiram in a complex with copper chloride, and from herein, is referred to as DSF [30, 31].

**Figure 6:**
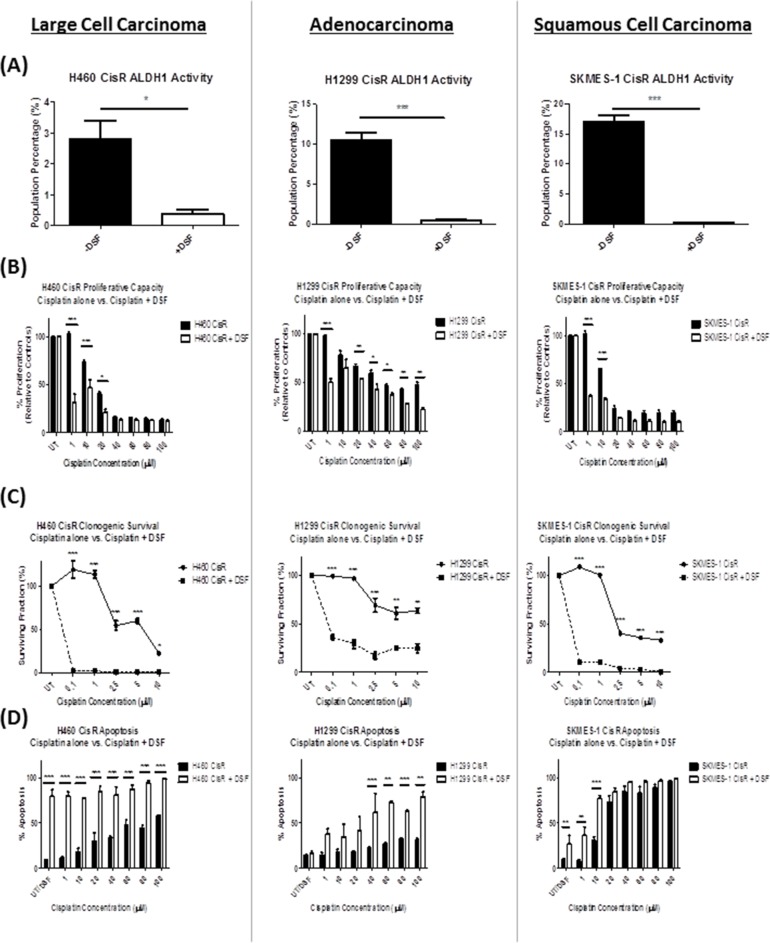
Repurposing of the drug, Disulfiram, re-sensitizes cisplatin resistant NSCLC cells to the cytotoxic effects of cisplatin Cisplatin resistant (CisR) sublines were treated with equimolar concentrations (0.25μM) of Disulfiram and copper chloride (DSF). **(A)** ALDH1 activity was measured by flow cytometry following treatment with DSF. DSF significantly reduced ALDH1 activity, as shown by a decrease in the percentage of ALDH1+ve cells in all three cisplatin resistant NSCLC sublines examined. **(B)** DSF in combination with cisplatin significantly decreased the cell proliferative capacity of H460, H1299 and SKMES-1 CisR sublines relative to cisplatin only controls. **(C)** Similarly, this DSF-cisplatin combination significantly reduced clonogenic survival of CisR sublines. **(D)** Flow cytometry analysis of Annexin V/PI demonstrated a significant increase in apoptotic cell death across all three CisR sublines. Data are shown from three independent experiments and are represented as Mean ± SEM (*p<0.05, **p<0.01, ***p<0.001).

Similar to that observed with the chemical inhibitor, DEAB, treatment of cisplatin resistant NSCLC sublines with DSF significantly inhibited ALDH1 activity (Figure 6A) in H460 CisR (p<0.05), H1299 CisR (p<0.001) and SKMES-1 (p<0.001) CisR sublines. Assessment of the proliferative capacity of these resistant cell lines in response to cisplatin alone and in combination with DSF, showed a significant increase in the sensitivity of these cell lines to the anti-proliferative and pro-apoptotic effects of cisplatin when treated in the presence of DSF (Figure 6B). While this effect was most significant at lower concentrations of cisplatin (1-20μM) in H460, H1299 and SKMES-1 CisR cell lines, DSF was also shown to significantly enhance the cytotoxicity of cisplatin in H1299 cells at higher concentrations of cisplatin (40-100μM) when treated in combination. This cytotoxicity of the Disulfiram/copper complex on cisplatin resistant lung cancer cells was also demonstrated in clonogenic survival studies, where DSF significantly reduced the survival ability of resistant clones at all concentrations of cisplatin examined (0.1-10μM) across all three CisR sublines (Figure 6C).

To determine the ability of DSF to re-sensitize CisR cells to cisplatin-induced cell death, apoptotic cell death was examined following treatment with increasing concentrations of cisplatin (0-100μM) alone, and in combination with DSF (Figure 6D). While DSF alone induced significant apoptosis of H460 and SKMES-1 CisR sublines relative to untreated cells, the addition of DSF to cisplatin at a range of concentrations examined, significantly increased cisplatin-mediated cell death. Of note, this was particularly significant in H460 CisR cells (p<0.001), in which case, DSF in combination with cisplatin at all concentrations (1-100μM) induced significant apoptosis, relative to cells treated with cisplatin and DSF alone. Interestingly, DSF alone did not induce apoptosis in H1299 CisR cells. However, treatment of this highly resistant subline with DSF in combination with increasing concentrations of cisplatin (40-100μM), significantly increased apoptotic cell death relative to cisplatin alone. These data highlight the potential clinical use of Disulfiram in circumventing or reversing cisplatin resistance in NSCLC cells, while depleting the stem-like subset of ALDH1+ve cells across all three histological subtypes.

## DISCUSSION

Since its introduction into clinical trials, cisplatin has revolutionized the treatment of several solid malignancies, including ovarian, testicular, head and neck and lung cancer [5]. Today, cisplatin forms the backbone of many combination therapeutic strategies and has been clinically tested with other chemotherapeutic agents including etoposide, gemcitabine and vinorelbine and targeted therapies such as erlotinib and gefitinib. While cisplatin represented a major landmark in the history of successful anti-cancer drugs, its success and efficacy are waning in the face of therapeutic resistance [32–37]. While the landscape of personalized medicine and targeted therapies is rapidly emerging and ever-changing, target identification, drug design, testing and approval takes time and is costly. In the interim and in the absence of specific driver mutations, improved efficacy of conventional cytotoxic agents is required in order to overcome current therapeutic hurdles such as drug resistance, in particular, for lung cancer patients. In the context of CSC biology, targeting CSC mechanisms alone, in isolation, may not be as effective as using a combination approach with conventional chemotherapeutic agents.

The maintenance and repair of many adult tissues is sustained by stem cells which reside at the top of the cellular hierarchy within tissues. The now widely accepted CSC hypothesis states that solid tumors also display a hierarchical organisation and contain CSCs that sustain tumor growth, survive anti-cancer therapy and promote relapse following therapy [38]. Since the discovery of CSCs in haematopoietic cancers and other solid tumors, little is known to date regarding the biology of lung CSCs. The existence of CSCs within the tumor cell population may explain the ineffectiveness of current treatments in eradicating tumor cells. While the aim of most therapies is to target the majority of cancer cells within the tumor population, residual lung CSCs can regenerate a cancer cell population resulting in tumor relapse in patients following chemotherapy. As such, there is an increasing need to identify and develop new therapeutic targets for specifically eradicating this cell population. While the marker profile of lung CSCs remains to be fully explored, the variety of markers reported to date, underlines the presence of different CSCs and progenitors within the various NSCLC tissues. It is now well established that specific CSCs within tumors are responsible for resistance to chemotherapeutic agents. Pre-clinical studies have established that CSCs that form part of the tumor cell population in a number of solid tumors are chemoresistant [39, 40]. In ovarian cancer, ALDH+ve CSCs have been consistently shown to exhibit increased chemoresistance, where the extent of the ALDH+ve subpopulation often correlates with acquired taxane and platinum resistance [41, 42].

Many stem-like cells commonly overexpress markers such as *NANOG*, *OCT-4*, *SOX-2*, *KLF-4* and *C-MYC*, where these genes play important roles in the regulation of self-renewal and tumorigenicity in CSC populations of several cancer types. The growing body of CSC research has also highlighted a number of CSC-specific markers that have been shown to display a stem-like phenotype, and include *ALDH1* and *CD133* [43, 44]. In order to identify a potential stemness phenotype within this NSCLC study, matched normal and tumor tissues of adenocarcinoma and squamous cell carcinoma subtypes were profiled for their expression of the aforementioned stemness and CSC-specific markers at the mRNA level. Significant up-regulation of a number of stem-associated markers across both adenocarcinoma and squamous cell carcinoma tissues confirmed the presence of a stem-like phenotype within this cohort of lung tumor tissues relative to matched normal lung tissue. Similar observations were reported by Zakaria *et al*., in the lung adenocarcinoma cell lines, A549 and H2170 compared to the normal bronchial epithelial cell line, PHBEC, highlighting the cancer stem cell hypothesis as an important avenue of interest in lung cancer [45]. Altered expression of these stem-associated markers was further validated in an *in vitro* model of cisplatin resistance using a panel of isogenic cisplatin resistant NSCLC sublines. In a study of oral squamous cell carcinomas (OSCC), CSC-like markers expressed in cisplatin resistant oral carcinomas such as *NANOG* and *OCT-4* became expanded during the acquisition of cisplatin resistance in OSCC. It was postulated based on these findings that overexpression of these stemness markers may promote cisplatin resistance in OSCCs that subsequently recur [46].

While the marker profile of lung CSCs remains to be fully explored, ALDH1 has emerged as a potential marker for CSCs in a number of solid tumors such as breast, head & neck, stomach, prostate, colon [23, 47–50] and has had increasing prominence as a stemness marker in lung cancer [26]. Our data show that acquired cisplatin resistance correlates with the expression of the ALDH+ve compartment in NSCLC models. Cisplatin resistant NSCLC sublines representing large cell, adenocarcinoma and squamous cell carcinoma subtypes displayed significantly greater ALDH1 activity compared to their corresponding parental controls. Moreover, we show that treatment of H460, H1299 and SKMES-1 cell lines with cisplatin resulted in the emergence of a significant, previously undetectable, ALDH1+ve subpopulation or the expansion of a pre-existing ALDH1+ve subpopulation. The emergence of this ALDH1+ve CSC population in both PT and CisR sublines corresponded with an increased resistance to cisplatin and upregulation of pluripotency markers, further implicating cisplatin as a contributing factor in the induction and expansion of a CSC population within tumors. Previous studies have reported the ability of cisplatin to select for a subset of drug-surviving cells with distinct CSC-like properties [16]. A quiescent subpopulation of glioma cells with CSC properties was shown to be implicated in tumor re-growth following treatment of glioblastomas with temozolomide [51]. Furthermore, long-term trastuzumab treatment enriched for a CSC population in breast cancer [52]. In more recent studies in head and neck squamous cell carcinoma (HNSCC), cisplatin enhanced the fraction of head and neck CSCs *in vivo*, despite the lack of a significant change in overall tumor volume [53]. Similarly, in ovarian cancer cells, following treatment with increasing concentrations of cisplatin, while there was a distinct dose-dependent decrease in the total number of viable cells, a significant increase in the percentage of ALDH+ve cells was observed, suggesting the chemoresistant nature of this ALDH+ve cell population and/or the induction of ALDH by cisplatin [54].

With respect to lung cancer, recent studies have demonstrated that treatment with paclitaxel induced a greater ALDH1+ve CSC population in H460 and H1299 lung cancer cell lines, while treatment with the selective CSC inhibitor, salinomycin, decreased the presence of the ALDH1+ve population and reduced the tumorsphere formation potential of these cells, but had no effect on the ALDH1-ve population [28]. Similarly, treatment with paclitaxel *in vivo* decreased tumor volume but increased the number of metastatic nodules, possibly due to the expansion of the ALDH1+ve tumor-initiating CSC population. Salinomycin alone did not reduce the size of the primary tumor but did decrease metastasis. In addition to cytotoxic agents, radiotherapy has also been shown to induce ALDH1+ve CSCs in A549 cells. Repeated exposure to 4Gy radiation was used to generate a radioresistant A549 subline which was shown to have greater proliferative and clonogenic capabilities when challenged with radiation compared to their radiosensitive counterparts. The emergence of an ALDH1+ve subpopulation correlated with the development of radioresistance and increasing doses of radiation [55]. Our findings of cisplatin-mediated induction of the CSC marker, ALDH1, in both parental and cisplatin resistant NSCLC cell lines, highlights the potential of cisplatin to select for, and expand, a CSC-like subpopulation of cells. These findings, together with those previously reported for other tumor types, support the concept that during the course of treatment with chemotherapy, cisplatin selects for a subset of drug-resistant ALDH1+ve stem cells, which may explain, at least in part, relapse of NSCLC patients following prolonged treatment with cisplatin.

Our data suggest that ALDH1+ve cells not only correlate with acquired cisplatin resistance but out-survive their ALDH1-ve counterparts during cisplatin therapy. In this study, we show that, relative to ALDH1-ve subpopulations isolated from chemoresistant NSCLC cell lines, the ALDH1+ve subsets have significantly increased proliferative and survival abilities when challenged with cisplatin. In addition to this observed increase in cisplatin resistance, an up-regulation of stemness genes and CSC makers was also shown. ALDH1 overexpression is associated with poor prognosis in NSCLC patients, where high ALDH1 expression was significantly associated with a more aggressive and advanced pathological grade and stage [27]. Furthermore, increased ALDH1 expression has been associated with increased metastasis in multiple cancers, including inflammatory breast cancer [56, 57] and has been shown to represent a tumor initiating population [58–61]. As such, we hypothesized that the ALDH1+ve cells were CSCs. Functionally CSCs are defined by their self-renewal, differentiation and tumorigenic potential. Our data demonstrate that ALDH1+ve cells, but not ALDH1-ve cells, are responsible for the production of the ALDH1+ve cisplatin resistant cell type. Only the ALDH1+ve cells had the ability to asymmetrically divide, where a single ALDH1+ve cell could give rise to a heterogenous progeny of both ALDH1+ve and ALDH1-ve cells in culture. Such findings show that only ALDH1+ve cells have the potential to self-renew and the capacity to differentiate into a unipotent lineage, which in turn, allows the propagation of the cisplatin resistant ALDH1+ve lineage of these cells. These findings are consistent with the current literature regarding ALDH1 as a CSC marker in NSCLC, and its role in resistance to molecular targeted therapies such as EGFR tyrosine kinase inhibitors [62].

To determine whether cisplatin resistant NSCLC cells presenting with high ALDH1 activity are also enriched with tumorigenic properties, ALDH1+ve and ALDH1-ve cell subpopulations isolated from H460 cisplatin resistant cells were studied in a human xenograft mouse model using NOD/SCID mice. We showed that both the ALDH1+ve and ALDH1-ve cell subsets could efficiently form tumors *in vivo* from low cell numbers. These data demonstrate that both ALDH1 subpopulations have tumorigenic capabilities. However, when taken together with the *in vitro* self-renewal and differentiation data, we have shown that only the ALDH1+ve CSCs are responsible for the propagation of the cisplatin resistant branch of NSCLC. Meanwhile, ALDH1-ve cell subsets propagate the more cisplatin sensitive branch of the NSCLC hierarchy. Similar findings have also been observed in a model of cisplatin resistant and sensitive CSC branches in an ovarian cancer hierarchy [63]. It is noted that the literature tends to reflect ALDH1+ve lung cancer cells as being more tumorigenic relative to ALDH1-ve cells. Sullivan et al [57] showed that ALDH1+ve and ALDH1-ve cells isolated from the lung cancer cell lines H358 and H1299, both generated tumors, where those derived from ALDH1+ve cells were significantly larger and grew faster compared to those formed from their ALDH1-ve counterparts. In ovarian cancer, chemoresistant ALDH1+ve subpopulations isolated from SKOV3 and HEY1 cell lines formed tumors in mice following injection with as little as 100 ALDH1+ve cells. Similar number of ALDH1 cells only rarely formed tumors [64]. Other studies report that while ALDH activity tends to enrich for CSCs when compared to their ALDH-ve counterparts, the opposite is also observed in a patient (tissue source) dependent manner [65]. With this in mind, the lung cancer cells employed in the study by Sullivan et al., H358 and H1299, are bronchioalveolar carcinoma and adenocarcinoma cell lines derived from metastatic sites, whereas the H460 cell line adopted in our study, was derived from a human large-cell carcinoma of the lung; a histotype defined by its lack of differentiated features. Together, these data support the emerging concept of histotype-specific CSC hierarchies.

Conventional anti-cancer therapies such as chemotherapy and radiotherapy have multiple limitations that ultimately result in treatment failure and tumor recurrence. Such agents are not selective, resulting in significant toxicities both locally and systemically. The development of drug resistance due to CSC expansion during treatment is a major clinical challenge. Therefore, the concept of targeting CSCs with novel agents potentially allows for increased specificity and efficiency in the treatment of solid tumors thereby preventing tumor relapse and enhancing patient survival. The development of novel anti-cancer drugs against various malignant tumors is both time-consuming and expensive and involves pre-clinical and clinical testing. Finding new uses for existing drugs, otherwise known as “repurposing”, may allow for potential new uses of the drug that are not consistent with known disease mechanisms and may lead to the discovery of new biological processes or disease pathways.

Disulfiram (Antabuse) has been widely used as a first-line drug in the treatment of alcoholism for the past 60 years [66]. Its primary pharmacological action is inhibition of aldehyde dehydrogenase (ALDH) which is responsible for converting acetaldehyde to acetate in the metabolism of alcohol. It is an irreversible pan-ALDH inhibitor and is known to inhibit all currently identified cytosolic and mitochondrial ALDH isoforms. More recently, accumulating evidence suggests that Disulfiram has significant anti-cancer activity against a number of cancer types such as prostate [67], breast [68], melanoma [69] and glioblastoma [70], both *in vitro* and *in vivo*. In a recent lung cancer study by Liu et al., [71] RNA interference and overexpression of ALDH isozymes suggested that ALDH1A1, which plays a key role in ALDH1-positive NSCLC stem cells, may be a target for the Disulfiram/CuCl_2_ complex, where DSF was shown to target ALDH1A1 and inhibit NSCLC recurrence driven by ALDH1-positive CSCs. It has been shown to enhance the cytotoxicity of several anti-cancer drugs and radiotherapy [72]. As Disulfiram is an FDA-approved drug, it represents an important drug for testing the proof-of-principle of ALDH inhibitors as CSC targeting agents in cisplatin resistant NSCLC. More importantly, Disulfiram has been safely used in cancer patients in combination with chemotherapy, suggesting that normal stem cells can tolerate such ALDH targeted therapies [73]. Its mechanism of action, pharmacokinetics, toxicity and tolerability are well known, and the drug is relatively non-toxic by itself at therapeutic doses. In addition, Disulfiram can be taken orally thereby making it convenient to administer. Unlike many chemotherapeutic agents, it can penetrate the blood-brain barrier and may therefore have an active effect on CNS metastases. To date, a number of clinical trials using Disulfiram have been initiated in lung cancer (NCT00312819), refractory solid tumors involving the liver (NCT00742911), metastatic melanoma (NCT00256230) and prostate cancer (NCT01118741).

Lung cancer has been poorly investigated in relation to the therapeutic benefits of Disulfiram. Recent results from a Phase IIb clinical trial in which Disulfiram was added to the chemotherapeutic treatment of metastatic NSCLC, showed that the combination of Disulfiram with cisplatin and vinorelbine was well-tolerated compared to chemotherapy alone and prolonged survival (10 vs 7.1 months) in newly diagnosed stage IV patients [74]. The data generated in this study using Disulfiram, support our hypothesis that specific targeting of the ALDH1+ve subpopulation within a resistant tumor cell population, re-sensitizes cells to the cytotoxic effects of cisplatin. Furthermore, while potentially enhancing the response of resistant lung tumor cells to cisplatin, Disulfiram may, at the same time, avoid further toxicities. Treatment of malignant pleural mesothelioma cell lines (H226, AB12 and H-Meso) with Disulfiram and copper resulted in suppressed cell growth and apoptosis. However, the cytotoxic effects of Disulfiram were not assessed in combination with conventional chemotherapeutic agents [75]. Disulfiram-copper combination therapy has been reported in the NSCLC adenocarcinoma cell lines, A549 and H2009. Treatment resulted in decreased cell viability and colony formation and increased cell cycle arrest in the G2/M phase. Disulfiram in combination with cisplatin decreased cell viability when compared to cisplatin alone [76]. To our knowledge, this study is the first of its kind whereby Disulfiram has been investigated in a model of cisplatin resistance across the spectrum of NSCLC subtypes.

Several studies have been reported that show elevated expression of ALDH1 in NSCLC patients. In a study by You et al [77], high gene expression of the ALDH1 isoform, ALDH1A1, correlated with better overall survival (OS) in adenocarcinoma patients when studied in a cohort of 1,926 NSCLC patients followed over a period of 20 years (p=0.039). These data demonstrating an association of ALDH1 mRNA with better prognosis were further supported in an exploratory and retrospective study [78] in NSCLC, which indicated that ALDH1 expression is associated with a more favourable outcome. These findings however, are in contrast to those by Jiang et al [26] which showed high ALDH1 expression was significantly associated with a more advanced pathologic grade and stage and conferred a poor clinical outcome for lung cancer patients, suggesting that ALDH1 plays an important role in the progression of this disease.

While studies examining whether expression of ALDH1 is linked to response to anti-cancer therapies are more limited, studies have examined cohorts of patients undergoing neoadjuvant chemotherapy, chemoradiotherapy or radiotherapy followed by complete surgical resection. In one study, the 5-year overall survival rate of patients with CD133-positive or ALDH1-positive specimens was significantly worse than that of patients with both CD133-negative and ALDH1-negative expression (44.9% vs. 90.0%, respectively; p=0.042) [79]. The expression of these CSC markers following chemoradiotherapy (CRT) correlated significantly with a poor prognosis in NSCLC patients. A multivariate analysis also identified expression of ALDH1 in NSCLC patients as a significant independent prognostic factor for disease-free survival [80]. The authors reported that the 5-year disease-free survival rate for patients with high ALDH1 expression levels in their cancer cells was significantly lower than those with low ALDH1 levels (47.3% vs. 21.5%, respectively; p=0.023). These data clearly indicate that CSC-related marker positivity may be assessed prior to chemotherapy-based interventions and could have prognostic value for patients with NSCLC who are treated with neoadjuvant therapy. While the majority of studies examining ALDH1 inhibitors such as DSF and/or DEAB alone [68, 70, 81, 82] and in combination with cisplatin [76, 83] have been carried out using *in vitro* models, the inhibitory effects of these ALDH1 agents on tumour growth in combination with chemotherapeutic agents such as cisplatin are warranted in clinically relevant tumour xenograft models. In one study, using a cell line-derived syngenic mouse model of breast cancer, DSF and radiation therapy significantly inhibited primary tumour growth and spontaneous lung metastasis [84].

## MATERIALS AND METHODS

### Ethics statement

This investigation was conducted in accordance with the ethical standards and according to the Declaration of Helsinki as well as national and international guidelines and has been approved by the authors’ institutional review board.

### Drugs

Cisplatin (Sigma-Aldrich) was dissolved in 0.15M NaCl. Aliquots were stored at −20°C for a maximum of 3 months and thawed immediately before use. Diethylaminobenzoaldehyde (DEAB), Disulfiram (DSF) and copper chloride (CuCl_2_) (Sigma-Aldrich) were dissolved in 95% (v/v) ethanol. Aliquots were stored at 4°C.

### Cell lines

The human large cell carcinoma cell line, NCI-H460 (hereafter referred to as H460) and its resistant variant were kindly donated by Dr Dean Fennell, Centre for Cancer Research and Cell Biology, Queen's University Belfast [86]. The human adenocarcinoma cell line, H1299, and its resistant subline were given as a gift from Dr Parviz Behnam-Motlagh, Department of Medical Biosciences, Umeå University, Sweden. The SKMES-1 squamous cell carcinoma cell line was purchased from the American Type Culture Collection (ATCC) (LGC Promochem, UK). Cisplatin resistant (CisR) sublines were generated from each original parental (PT) cell line by continuous exposure to cisplatin, as previously described [29]. Briefly, cells were treated with cisplatin (IC_50_) for 72hrs, after which time cisplatin-containing media was removed and cells were allowed to recover for a further 72hrs. This development period was carried out for 6 months, after which time IC_50_ concentrations were reassessed and used as a maintenance dose for a further 6 months. H460 cells were grown in Roswell Park Memorial Institute (RPMI-1640) media. H1299 and SKMES-1 cells were maintained in Eagle's Minimum Essential Medium (EMEM) supplemented with 2mM L-glutamine and 1× non-essential amino acids (NEAA). For all cell lines, media was supplemented with 10% heat-inactivated foetal bovine serum (FBS), penicillin (100U/ml) and streptomycin (100μg/ml) (Lonza, UK). Cell lines were previously tested and authenticated using the PowerPlex^®^ 16 HS System (Source BioScience, UK), grown as monolayer cultures and maintained in a humidified atmosphere of 5% CO_2_ at 37°C.

### Patient samples

Informed consent was obtained from all patients prior to tissue procurement. Tissue samples (matched normal and tumor lung) from resected NSCLC patients were obtained from the Lung Cancer Biobank at St. James's Hospital, Dublin. All studies carried out were performed with the approval of our local Institutional Review Board (IRB), the St. James's Hospital and Federated Dublin Voluntary Hospitals Joint Research Ethics Committee.

### Reverse transcriptase polymerase chain reaction (RT-PCR)

Total RNA was extracted using TRI Reagent (Molecular Research Center, USA) according to manufacturer's instructions. cDNA was transcribed from 1μg of total RNA using SuperScript III reverse transcriptase (Invitrogen) according to the manufacturer's instructions. Gene expression (mRNA) analysis of stem cell and CSC markers was carried out by RT-PCR using the following primers: *NANOG* (FWD 5′-TTGGAGC CTAATCAGCGAGGT-3′, REV 5′-GCCTCCCAATCCCA AACAATA-3′), *OCT-4* (FWD 5′-ATTCAGCCAAAC GACCATCT-3′, REV 5′-GTTTTCTTACTAGTCAC GTGCGG-3′), *SOX-2* (FWD 5′-GGAGCTTTGCACG AAGTTTG-3′, REV 5′-GGAAAGTTGGGATCGA ACAA-3′), *KLF4* (FWD 5′-CACACTTGTGATTACGC GGG-3′, REV 5′-CCCGTGTGTTTACGGTAGTGC-3′), *C-MYC* (FWD 5′-CCTCGGATTCTCTGCTCTCCTC-3′, REV 5′-AGGTTTGCTGTGGCCTCCAG-3′), *ALDH1* (FWD 5′-GCCATAACAATCTCCTCTGCT-3′, REV 5′-CATGGAAACCGTACTCTCCC-3′) and *CD133* (FWD 5′-GAGAAAGTGGCATCGTGCAA-3′, REV 5′-CA CGTCCTCCGAATCCATTC-3′). *β-actin* was used as an endogenous loading control (FWD 5′-TGTTTGAGA CCTTCAACACCC-3′, REV 5′-AGCACTGTGTTG GCGTACAG-3′). Template cDNA was initially denatured at 95°C for 5mins, followed by 35-40 amplification cycles consisting of denaturation at 95°C for 1min, primer-specific annealing for 1min and extension at 72°C for 1min. Cycles were followed by an elongation step of 72°C for 10mins. PCR products were resolved on 2% agarose gels containing ethidium bromide. Images were acquired using the Fusion FX imaging system (Vilber Lourmat, Germany). Product quantification was performed using ImageJ densitometry software. Gene expression was normalized to endogenous β-actin controls and was expressed as fold-change.

### Cell proliferation

Cell proliferation was measured using the Cell Proliferation BrdU ELISA (Roche Diagnostics Ltd., UK), according to manufacturer's instructions. Briefly, cells (H460, H1299 and SKMES-1) were seeded at 2.5 × 10^3^/well in a 96-well plate. Following overnight incubation, cells were treated for 72hr with cisplatin (0-100μM) alone, or in combination with DEAB (15μM) or equimolar concentrations of Disulfiram (0.25μM) and copper chloride (0.25μM) (DSF/CuCl_2_). Absorbance was recorded at 450 nm and sensitivity to cisplatin was calculated based on the percentage cell proliferation relative to untreated controls, which were set at 100%.

### Aldefluor assay and cell sorting

The Aldefluor assay (Stem Cell Technologies, Canada) was used to identify and isolate cell populations with ALDH1 enzymatic activity. The assay was carried out according to manufacturer's instructions. Briefly, cells (5 × 10^5^) were suspended in Aldefluor assay buffer containing activated Aldefluor reagent, BODIPY-aminoacetaldehyde (BAAA) for 45 min. The Aldefluor reagent is a fluorescent non-toxic ALDH1 substrate that freely diffuses into intact viable cells. In the presence of ALDH1, BAAA is converted to BOPIDY-aminoacetate (BAA), which is retained within the cells expressing ALDH1. A specific ALDH1 inhibitor, DEAB, was used to inhibit the BAAA-BAA conversion and acts as an internal negative control for background fluorescence. The brightly fluorescent ALDH1+ve cells were detected using the green fluorescence channel (520-540nm). ALDH1 activity was measured using a CyAn^TM^ ADP flow cytometer (Dako, USA), while ALDH1+ve and ALDH1-ve fractions were sorted using a MoFlo^TM^ XDP high speed cell sorter (Beckman Coulter, USA).

### Clonogenic survival

The survival of NSCLC cells, when challenged with cisplatin, was measured using the clonogenic assay [87]. Cells were seeded at optimal cell densities and allowed to adhere overnight at 37°C. Cells were treated with increasing concentrations of cisplatin (0-10μM) for 72hrs alone, or in combination with DEAB (15μM) or DSF/CuCl_2_ (0.25μM/0.25μM), after which time, culture media was removed and replaced with fresh treatment-free media and re-incubated for a further 10 days. Colonies were fixed and stained with 25% (v/v) methanol, 0.05% (w/v) crystal violet for 30 mins. Residual stain was removed by rinsing wells gently with tap water. Colonies were counted using the ColCount^TM^ colony counter (Oxford Optronix Ltd, Oxford, UK). Plating efficiencies (PE) were calculated using the formula: PE = Number of colonies/Number of cells seeded. The percentage surviving fraction (SF) was calculated using the formula: SF = (PE treated colonies/PE untreated) ×100.

### Asymmetric division

Cells were stained using the Aldefluor assay and sorted using the MoFlo™ XDP high-speed cell sorter (Beckman Coulter). ALDH1+ve and ALDH1-ve cells were sorted and plated as a single cell per well of a 96-well plate containing media. Each well was supplemented with fresh media every 3 to 4 days. At week 2, wells were examined under an inverted phase-contrast Nikon Eclipse TS100/100-F microscope. Wells containing visible colonies were maintained in culture until confluent, at which point, cells were trypsinized and plated into wells of a 24-well plate. Cells were further sub-cultured into 25cm^2^ flasks. Cells were trypsinized and re-stained using the Aldefluor assay to reassess the ALDH1 cell subsets, ALDH1+ve, ALDH1-ve and ALDH1+/−.

### Apoptosis

Apoptosis was measured by flow cytometry using dual Annexin-V and propidium iodide (PI) staining [88]. Cells (1 × 10^5^ cells) were seeded in 6-well plates and allowed to adhere overnight. Cells were treated with increasing concentrations of cisplatin (0-100μM) alone, or in combination with DEAB (15μM) or DSF/CuCl_2_ (0.25μM/0.25μM) for 48hrs. Untreated control cells were treated with media only. Following treatment, floating and adhered cells were collected, transferred to FACS tubes and placed on ice. Cells were pelleted by centrifugation and washed in 1ml of 1X Annexin binding buffer (BB), pelleted, and re-suspended in 200μl BB. A volume of 2μl Annexin V-FITC (IQ Products) was added to each test sample and incubated at 4°C for 20min. After this time, 1ml of 1X BB was added to each tube and cells pelleted by centrifugation. Immediately before FACS analysis, cells were re-suspended in 400μl of 1X BB containing 1μg/ml PI (Invitrogen). Apoptotic cells were measured using a CyAn™ ADP flow cytometer (Dako, USA).

### *In vivo* tumor formation assay

Female 7-9 week old NOD SCID (NOD.CB17-Prkdcscid/NCrCrl) mice were obtained from Charles River Laboratories. NOD/SCID mice (n=4 per group) were subcutaneously inoculated with ALDH1+ve and ALDH1-ve H460 CisR cell fractions at a density of 1×10^3^ cells/mouse within a Matrigel plug (Corning, USA). Tumor volumes were recorded thrice weekly using digital callipers. Tumor volumes were calculated using the modified ellipsoid formula [1/2 (Length x Width^2^)] [89]. Experimental endpoints were defined as a tumor volume of 500mm^3^ or 90 days post-inoculation, at which point animals were sacrificed and tumors harvested. All animal experiments were approved by the Ethics Board of Trinity College Dublin and carried out under a licence granted by the Health Products Regulatory Authority.

### Statistical analysis

Analysis between groups was carried out using analysis of variance (ANOVA). Statistical comparison of two means was carried out using an unpaired, two-tailed Student's t-test. Significance was defined as p≤0.05. Data is graphically represented as mean ± standard error of the mean (SEM). Statistical analysis of *in vivo* data was carried out using the non-parametric Mann-Whitney test. All data were analysed using GraphPad Prism^TM^ (version 5) statistical software.

## CONCLUSION

This study set out to investigate the potential targeting of ALDH1 in cisplatin resistant NSCLC using *in vitro* and *in vivo* models and to further explore CSC-mediated mechanisms of resistance. While our data demonstrate an important role for ALDH1 as a stemness marker in chemoresistant lung cancer cells, inhibition of its activity and subsequent re-sensitization to the cytotoxic effects of cisplatin via chemical and pharmacological inhibition using DEAB and Disulfiram, respectively, demonstrates the potential use of these agents as a future strategy in targeting subsets of CSCs in cisplatin resistant lung tumors. Further molecular studies characterizing lung CSCs will yield additional information regarding the behaviour of these stem cell subsets, in addition to identifying novel targeted therapies. Of interest, ALDH1A1^−/−^ mice are viable [85], suggesting that ALDH1 inhibition is unlikely to have detrimental effects on normal tissue stem cells. While multiple isoforms of ALDH do exist, and are differentially expressed in different cancer types, ALDH1 is one potential target for therapy in the context of cisplatin resistant lung cancer. The availability of the well-tolerated, FDA-approved drug, Disulfiram, may give cisplatin a new lease of life in the treatment of previously resistant lung tumors.

## Abbreviations

ALDH: aldehyde dehydrogenase
NSCLC: non-small cell lung cancer
SCLC: small cell lung cancer
DEAB: diethylaminobenzaldehyde
CSC: cancer stem cell
PT: parental
CisR: cisplatin resistant
RT-PCR: reverse transcriptase polymerase chain reaction
DSF: Disulfiram
OSCC: oral squamous cell carcinoma
CuCl_2_: copper chloride

## References

[1] Jemal A, Bray F, Center MM, Ferlay J, Ward E, Forman D (2011). Global cancer statistics. CA Cancer J Clin.

[2] Ferlay J, Soerjomataram I, Dikshit R, Eser S, Mathers C, Rebelo M, Parkin DM, Forman D, Bray F (2015). Cancer incidence and mortality worldwide: sources, methods and major patterns in GLOBOCAN 2012. Int J Cancer.

[3] Goldstraw P, Chansky K, Crowley J, Rami-Porta R, Asamura H, Eberhardt WE, Nicholson AG, Groome P, Mitchell A, Bolejack V;, International Association for the Study of Lung Cancer Staging and Prognostic Factors Committee, Advisory Boards, and Participating Institutions, International Association for the Study of Lung Cancer Staging and Prognostic Factors Committee Advisory Boards and Participating Institutions (2016). The IASLC Lung Cancer Staging Project: proposals for revision of the TNM stage groupings in the forthcoming (Eighth) edition of the TNM classification for lung cancer. J Thorac Oncol.

[4] Ferlay J, Steliarova-Foucher E, Lortet-Tieulent J, Rosso S, Coebergh JW, Comber H, Forman D, Bray F (2013). Cancer incidence and mortality patterns in Europe: estimates for 40 countries in 2012. Eur J Cancer.

[5] Kelland L (2007). The resurgence of platinum-based cancer chemotherapy. Nat Rev Cancer.

[6] Reya T, Morrison SJ, Clarke MF, Weissman IL (2001). Stem cells, cancer, and cancer stem cells. Nature.

[7] Pardal R, Clarke MF, Morrison SJ (2003). Applying the principles of stem-cell biology to cancer. Nat Rev Cancer.

[8] Kreso A, Dick JE (2014). Evolution of the cancer stem cell model. Cell Stem Cell.

[9] Matsuda S, Yan T, Mizutani A, Sota T, Hiramoto Y, Prieto-Vila M, Chen L, Satoh S, Kudoh T, Kasai T, Murakami H, Fu L, Salomon DS (2014). Cancer stem cells maintain a hierarchy of differentiation by creating their niche. Int J Cancer.

[10] Morrison SJ, Kimble J (2006). Asymmetric and symmetric stem-cell divisions in development and cancer. Nature.

[11] Cocola C, Sanzone S, Astigiano S, Pelucchi P, Piscitelli E, Vilardo L, Barbieri O, Bertoli G, Reinbold RA, Zucchi I (2008). A rat mammary gland cancer cell with stem cell properties of self-renewal and multi-lineage differentiation. Cytotechnology.

[12] Serrano D, Bleau AM, Fernandez-Garcia I, Fernandez-Marcelo T, Iniesta P, Ortiz-de-Solorzano C, Calvo A (2011). Inhibition of telomerase activity preferentially targets aldehyde dehydrogenase-positive cancer stem-like cells in lung cancer. Mol Cancer.

[13] Liu YP, Yang CJ, Huang MS, Yeh CT, Wu AT, Lee YC, Lai TC, Lee CH, Hsiao YW, Lu J, Shen CN, Lu PJ, Hsiao M (2013). Cisplatin selects for multidrug-resistant CD133+ cells in lung adenocarcinoma by activating Notch signaling. Cancer Res.

[14] Wintzell M, Lofstedt L, Johansson J, Pedersen AB, Fuxe J, Shoshan M (2012). Repeated cisplatin treatment can lead to a multiresistant tumor cell population with stem cell features and sensitivity to 3-bromopyruvate. Cancer Biol Ther.

[15] Hamilton G, Olszewski U (2013). Chemotherapy-induced enrichment of cancer stem cells in lung cancer. J Bioanal Biomed.

[16] Levina V, Marrangoni AM, DeMarco R, Gorelik E, Lokshin AE (2008). Drug-selected human lung cancer stem cells: cytokine network, tumorigenic and metastatic properties. PLoS One.

[17] Calcagno AM, Salcido CD, Gillet JP, Wu CP, Fostel JM, Mumau MD, Gottesman MM, Varticovski L, Ambudkar SV (2010). Prolonged drug selection of breast cancer cells and enrichment of cancer stem cell characteristics. J Natl Cancer Inst.

[18] Sharma SV, Lee DY, Li B, Quinlan MP, Takahashi F, Maheswaran S, McDermott U, Azizian N, Zou L, Fischbach MA, Wong KK, Brandstetter K, Wittner B (2010). A chromatin-mediated reversible drug-tolerant state in cancer cell subpopulations. Cell.

[19] Yoshida A, Hsu LC, Dave V (1992). Retinal oxidation activity and biological role of human cytosolic aldehyde dehydrogenase. Enzyme.

[20] Kastan MB, Schlaffer E, Russo JE, Colvin OM, Civin CI, Hilton J (1990). Direct demonstration of elevated aldehyde dehydrogenase in human hematopoietic progenitor cells. Blood.

[21] Hsu LC, Chang WC, Hoffmann I, Duester G (1999). Molecular analysis of two closely related mouse aldehyde dehydrogenase genes: identification of a role for Aldh1, but not Aldh-pb, in the biosynthesis of retinoic acid. Biochem J.

[22] Armstrong L, Stojkovic M, Dimmick I, Ahmad S, Stojkovic P, Hole N, Lako M (2004). Phenotypic characterization of murine primitive hematopoietic progenitor cells isolated on basis of aldehyde dehydrogenase activity. Stem Cells.

[23] Ginestier C, Hur MH, Charafe-Jauffret E, Monville F, Dutcher J, Brown M, Jacquemier J, Viens P, Kleer CG, Liu S, Schott A, Hayes D, Birnbaum D (2007). ALDH1 is a marker of normal and malignant human mammary stem cells and a predictor of poor clinical outcome. Cell Stem Cell.

[24] Clay MR, Tabor M, Owen JH, Carey TE, Bradford CR, Wolf GT, Wicha MS, Prince ME (2010). Single-marker identification of head and neck squamous cell carcinoma cancer stem cells with aldehyde dehydrogenase. Head Neck.

[25] Li T, Su Y, Mei Y, Leng Q, Leng B, Liu Z, Stass SA, Jiang F (2010). ALDH1A1 is a marker for malignant prostate stem cells and predictor of prostate cancer patients’ outcome. Lab Invest.

[26] Jiang F, Qiu Q, Khanna A, Todd NW, Deepak J, Xing L, Wang H, Liu Z, Su Y, Stass SA, Katz RL (2009). Aldehyde dehydrogenase 1 is a tumor stem cell-associated marker in lung cancer. Mol Cancer Res.

[27] Okudela K, Woo T, Mitsui H, Suzuki T, Tajiri M, Sakuma Y, Miyagi Y, Tateishi Y, Umeda S, Masuda M, Ohashi K (2013). Downregulation of ALDH1A1 expression in non-small cell lung carcinomas-its clinicopathologic and biological significance. Int J Clin Exp Pathol.

[28] Larzabal L, El-Nikhely N, Redrado M, Seeger W, Savai R, Calvo A (2013). Differential effects of drugs targeting cancer stem cell (CSC) and non-CSC populations on lung primary tumors and metastasis. PLoS One.

[29] Barr MP, Gray SG, Hoffmann AC, Hilger RA, Thomale J, O'Flaherty JD, Fennell DA, Richard D, O'Leary JJ, O'Byrne KJ (2013). Generation and characterisation of Cisplatin-resistant non-small cell lung cancer cell lines displaying a stem-like signature. PLoS One.

[30] Iljin K, Ketola K, Vainio P, Halonen P, Kohonen P, Fey V, Grafström RC, Perälä M, Kallioniemi O (2009). High-throughput cell-based screening of 4910 known drugs and drug-like small molecules identifies disulfiram as an inhibitor of prostate cancer cell growth. Clin Cancer Res.

[31] Wang F, Zhai S, Liu X, Li L, Wu S, Dou QP, Yan B (2011). A novel dithiocarbamate analogue with potentially decreased ALDH inhibition has copper-dependent proteasome-inhibitory and apoptosis-inducing activity in human breast cancer cells. Cancer Lett.

[32] Wozniak AJ, Crowley JJ, Balcerzak SP, Weiss GR, Spiridonidis CH, Baker LH, Albain KS, Kelly K, Taylor SA, Gandara DR, Livingston RB (1998). Randomized trial comparing cisplatin with cisplatin plus vinorelbine in the treatment of advanced non-small-cell lung cancer: a Southwest Oncology Group study. J Clin Oncol.

[33] Gatzemeier U, Pluzanska A, Szczesna A, Kaukel E, Roubec J, De_Rosa F, Milanowski J, Karnicka-Mlodkowski H, Pesek M, Serwatowski P, Ramlau R, Janaskova T, Vansteenkiste J (2007). Phase III study of erlotinib in combination with cisplatin and gemcitabine in advanced non-small-cell lung cancer: the Tarceva Lung Cancer Investigation Trial. J Clin Oncol.

[34] Giaccone G, Herbst RS, Manegold C, Scagliotti G, Rosell R, Miller V, Natale RB, Schiller JH, Von_Pawel J, Pluzanska A, Gatzemeier U, Grous J, Ochs JS (2004). Gefitinib in combination with gemcitabine and cisplatin in advanced non-small-cell lung cancer: a phase III trial-INTACT 1. J Clin Oncol.

[35] Siddik ZH (2003). Cisplatin: mode of cytotoxic action and molecular basis of resistance. Oncogene.

[36] Gore ME, Fryatt I, Wiltshaw E, Dawson T, Robinson BA, Calvert AH (1989). Cisplatin/carboplatin cross-resistance in ovarian cancer. Br J Cancer.

[37] Fennell DA, Summers Y, Cadranel J, Benepal T, Christoph DC, Lal R, Das M, Maxwell F, Visseren-Grul C, Ferry D (2016). Cisplatin in the modern era: the backbone of first-line chemotherapy for non-small cell lung cancer. Cancer Treat Rev.

[38] Beck B, Blanpain C (2013). Unravelling cancer stem cell potential. Nat Rev Cancer.

[39] Chen K, Huang YH, Chen JL (2013). Understanding and targeting cancer stem cells: therapeutic implications and challenges. Acta Pharmacol Sin.

[40] Sarvi S, Mackinnon AC, Avlonitis N, Bradley M, Rintoul RC, Rassl DM, Wang W, Forbes SJ, Gregory CD, Sethi T (2014). CD133+ cancer stem-like cells in small cell lung cancer are highly tumorigenic and chemoresistant but sensitive to a novel neuropeptide antagonist. Cancer Res.

[41] Mizuno T, Suzuki N, Makino H, Furui T, Morii E, Aoki H, Kunisada T, Yano M, Kuji S, Hirashima Y, Arakawa A, Nishio S, Ushijima K (2015). Cancer stem-like cells of ovarian clear cell carcinoma are enriched in the ALDH-high population associated with an accelerated scavenging system in reactive oxygen species. Gynecol Oncol.

[42] Duester G (1998). Alcohol dehydrogenase as a critical mediator of retinoic acid synthesis from vitamin A in the mouse embryo. J Nutr.

[43] Seigel GM, Campbell LM, Narayan M, Gonzalez-Fernandez F (2005). Cancer stem cell characteristics in retinoblastoma. Mol Vis.

[44] Singh SK, Clarke ID, Terasaki M, Bonn VE, Hawkins C, Squire J, Dirks PB (2003). Identification of a cancer stem cell in human brain tumors. Cancer Res.

[45] Zakaria N, Yusoff NM, Zakaria Z, Lim MN, Baharuddin PJ, Fakiruddin KS, Yahaya B (2015). Human non-small cell lung cancer expresses putative cancer stem cell markers and exhibits the transcriptomic profile of multipotent cells. BMC Cancer.

[46] Tsai LL, Yu CC, Chang YC, Yu CH, Chou MY (2011). Markedly increased Oct4 and Nanog expression correlates with cisplatin resistance in oral squamous cell carcinoma. J Oral Pathol Med.

[47] Li XS, Xu Q, Fu XY, Luo WS (2014). ALDH1A1 overexpression is associated with the progression and prognosis in gastric cancer. BMC Cancer.

[48] van_den_Hoogen C, van_der_Horst G, Cheung H, Buijs JT, Lippitt JM, Guzmán-Ramírez N, Hamdy FC, Eaton CL, Thalmann GN, Cecchini MG, Pelger RC, van_der_Pluijm G (2010). High aldehyde dehydrogenase activity identifies tumor-initiating and metastasis-initiating cells in human prostate cancer. Cancer Res.

[49] Shenoy A, Butterworth E, Huang EH (2012). ALDH as a marker for enriching tumorigenic human colonic stem cells. Methods Mol Biol.

[50] Douville J, Beaulieu R, Balicki D (2009). ALDH1 as a functional marker of cancer stem and progenitor cells. Stem Cells Dev.

[51] Chen J, Li Y, Yu TS, McKay RM, Burns DK, Kernie SG, Parada LF (2012). A restricted cell population propagates glioblastoma growth after chemotherapy. Nature.

[52] Korkaya H, Kim GI, Davis A, Malik F, Henry NL, Ithimakin S, Quraishi AA, Tawakkol N, D'Angelo R, Paulson AK, Chung S, Luther T, Paholak HJ (2012). Activation of an IL6 inflammatory loop mediates trastuzumab resistance in HER2+breast cancer by expanding the cancer stem cell population. Mol Cell.

[53] Nör C, Zhang Z, Warner KA, Bernardi L, Visioli F, Helman JI, Roesler R, Nör JE (2014). Cisplatin induces Bmi-1 and enhances the stem cell fraction in head and neck cancer. Neoplasia.

[54] Silva IA, Bai S, McLean K, Yang K, Griffith K, Thomas D, Ginestier C, Johnston C, Kueck A, Reynolds RK, Wicha MS, Buckanovich RJ (2011). Aldehyde dehydrogenase in combination with CD133 defines angiogenic ovarian cancer stem cells that portend poor patient survival. Cancer Res.

[55] Mihatsch J, Toulany M, Bareiss PM, Grimm S, Lengerke C, Kehlbach R, Rodemann HP (2011). Selection of radioresistant tumor cells and presence of ALDH1 activity in vitro. Radiother Oncol.

[56] Charafe-Jauffret E, Ginestier C, Lovino F, Tarpin C, Diebel M, Esterni B, Houvenaeghel G, Extra JM, Bertucci F, Jacquemier J, Xerri L, Dontu G, Stassi G (2010). Aldehyde dehydrogenase 1-positive cancer stem cells mediate metastasis and poor clinical outcome in inflammatory breast cancer. Clin Cancer Res.

[57] Sullivan JP, Spinola M, Dodge M, Raso MG, Behrens C, Gao B, Schuster K, Shao C, Larsen JE, Sullivan LA, Honorio S, Xie Y, Scaglioni PP (2010). Aldehyde dehydrogenase activity selects for lung adenocarcinoma stem cells dependent on notch signaling. Cancer Res.

[58] Moreb JS (2008). Aldehyde dehydrogenase as a marker for stem cells. Curr Stem Cell Res Ther.

[59] Croker AK, Goodale D, Chu J, Postenka C, Hedley BD, Hess DA, Allan AL (2009). High aldehyde dehydrogenase and expression of cancer stem cell markers selects for breast cancer cells with enhanced malignant and metastatic ability. J Cell Mol Med.

[60] Carpentino JE, Hynes MJ, Appelman HD, Zheng T, Steindler DA, Scott EW, Huang EH (2009). Aldehyde dehydrogenase-expressing colon stem cells contribute to tumorigenesis in the transition from colitis to cancer. Cancer Res.

[61] Ucar D, Cogle CR, Zucali JR, Ostmark B, Scott EW, Zori R, Gray BA, Moreb JS (2009). Aldehyde dehydrogenase activity as a functional marker for lung cancer. Chem Biol Interact.

[62] Huang CP, Tsai MF, Chang TH, Tang WC, Chen SY, Lai HH, Lin TY, Yang JC, Yang PC, Shih JY, Lin SB (2013). ALDH-positive lung cancer stem cells confer resistance to epidermal growth factor receptor tyrosine kinase inhibitors. Cancer Lett.

[63] Gasch C, Ffrench B, O'Leary JJ, Gallagher MF (2017). Catching moving targets: cancer stem cell hierarchies, therapy-resistance & considerations for clinical intervention. Mol Cancer.

[64] Landen CN, Goodman B, Katre AA, Steg AD, Nick AM, Stone RL, Miller LD, Mejia PV, Jennings NB, Gershenson DM, Bast RC, Coleman RL, Lopez-Berestein G (2010). Targeting aldehyde dehydrogenase cancer stem cells in ovarian cancer. Mol Cancer Ther.

[65] Stewart JM, Shaw PA, Gedye C, Bernardini MQ, Neel BG, Ailles LE (2011). Phenotypic heterogeneity and instability of human ovarian tumor-initiating cells. Proc Natl Acad Sci U S A.

[66] Johansson B (1992). A review of the pharmacokinetics and pharmacodynamics of disulfiram and its metabolites. Acta Psychiatr Scand Suppl.

[67] Lin J, Haffner MC, Zhang Y, Lee BH, Brennen WN, Britton J, Kachhap SK, Shim JS, Liu JO, Nelson WG, Yegnasubramanian S, Carducci MA (2011). Disulfiram is a DNA demethylating agent and inhibits prostate cancer cell growth. Prostate.

[68] Zhang H, Chen D, Ringler J, Chen W, Cui QC, Ethier SP, Dou QP, Wu G (2010). Disulfiram treatment facilitates phosphoinositide 3-kinase inhibition in human breast cancer cells in vitro and in vivo. Cancer Res.

[69] Morrison BW, Doudican NA, Patel KR, Orlow SJ (2010). Disulfiram induces copper-dependent stimulation of reactive oxygen species and activation of the extrinsic apoptotic pathway in melanoma. Melanoma Res.

[70] Liu P, Brown S, Goktug T, Channathodiyil P, Kannappan V, Hugnot JP, Guichet PO, Bian X, Armesilla AL, Darling JL, Wang W (2012). Cytotoxic effect of disulfiram/copper on human glioblastoma cell lines and ALDH-positive cancer-stem-like cells. Br J Cancer.

[71] Liu X, Wang L, Cui W, Yuan X, Lin L, Cao Q, Wang N, Li Y, Guo W, Zhang X, Wu C, Yang J (2016). Targeting ALDH1A1 by disulfiram/copper complex inhibits non-small cell lung cancer recurrence driven by ALDH-positive cancer stem cells. Oncotarget.

[72] Rae C, Tesson M, Babich JW, Boyd M, Sorensen A, Mairs RJ (2013). The role of copper in disulfiram-induced toxicity and radiosensitization of cancer cells. J Nucl Med.

[73] Verma S, Stewart DJ, Maroun JA, Nair RC (1990). A randomized phase II study of cisplatin alone versus cisplatin plus disulfiram. Am J Clin Oncol.

[74] Nechushtan H, Hamamreh Y, Nidal S, Gotfried M, Baron A, Shalev YI, Nisman B, Peretz T, Peylan-Ramu N (2015). A phase IIb trial assessing the addition of disulfiram to chemotherapy for the treatment of metastatic non-small cell lung cancer. Oncologist.

[75] Cheriyan VT, Wang Y, Muthu M, Jamal S, Chen D, Yang H, Polin LA, Tarca AL, Pass HI, Dou QP, Sharma S, Wali A, Rishi AK (2014). Disulfiram suppresses growth of the malignant pleural mesothelioma cells in part by inducing apoptosis. PLoS One.

[76] Duan L, Shen H, Zhao G, Yang R, Cai X, Zhang L, Jin C, Huang Y (2014). Inhibitory effect of Disulfiram/copper complex on non-small cell lung cancer cells. Biochem Biophys Res Commun.

[77] You Q, Guo H, Xu D (2015). Distinct prognostic values and potential drug targets of ALDH1 isoenzymes in non-small-cell lung cancer. Drug Des Devel Ther.

[78] Dimou A, Neumeister V, Agarwal S, Anagnostou V, Syrigos K, Rimm DL (2012). Measurement of aldehyde dehydrogenase 1 expression defines a group with better prognosis in patients with non-small cell lung cancer. Am J Pathol.

[79] Shien K, Toyooka S, Ichimura K, Soh J, Furukawa M, Maki Y, Muraoka T, Tanaka N, Ueno T, Asano H, Tsukuda K, Yamane M, Oto T (2012). Prognostic impact of cancer stem cell-related markers in non-small cell lung cancer patients treated with induction chemoradiotherapy. Lung Cancer.

[80] Zenke Y, Ishii G, Ohe Y, Kaseda K, Yoshida T, Matsumoto S, Umemura S, Yoh K, Niho S, Goto K, Ohmatsu H, Kuwata T, Nagai K (2013). Aldehyde dehydrogenase 1 expression in cancer cells could have prognostic value for patients with non-small cell lung cancer who are treated with neoadjuvant therapy: identification of prognostic microenvironmental factors after chemoradiation. Pathol Int.

[81] Chiba T, Suzuki E, Yuki K, Zen Y, Oshima M, Miyagi S, Saraya A, Koide S, Motoyama T, Ogasawara S, Ooka Y, Tawada A, Nakatsura T (2014). Disulfiram eradicates tumor-initiating hepatocellular carcinoma cells in ROS-p38 MAPK pathway-dependent and-independent manners. PLoS One.

[82] Moreb JS, Ucar D, Han S, Amory JK, Goldstein AS, Ostmark B, Chang LJ (2012). The enzymatic activity of human aldehyde dehydrogenases 1A2 and 2 (ALDH1A2 and ALDH2) is detected by Aldefluor, inhibited by diethylaminobenzaldehyde and has significant effects on cell proliferation and drug resistance. Chem Biol Interact.

[83] Liu P, Kumar IS, Brown S, Kannappan V, Tawari PE, Tang JZ, Jiang W, Armesilla AL, Darling JL, Wang W (2013). Disulfiram targets cancer stem-like cells and reverses resistance and cross-resistance in acquired paclitaxel-resistant triple-negative breast cancer cells. Br J Cancer.

[84] Wang Y, Li W, Patel SS, Cong J, Zhang N, Sabbatino F, Liu X, Qi Y, Huang P, Lee H, Taghian A, Li JJ, DeLeo AB (2014). Blocking the formation of radiation-induced breast cancer stem cells. Oncotarget.

[85] Tomita H, Tanaka K, Tanaka T, Hara A (2016). Aldehyde dehydrogenase 1A1 in stem cells and cancer. Oncotarget.

[86] Crawford N, Chacko AD, Savage KI, McCoy F, Redmond K, Longley DB, Fennell DA (2011). Platinum resistant cancer cells conserve sensitivity to BH3 domains and obatoclax induced mitochondrial apoptosis. Apoptosis.

[87] Franken NA, Rodermond HM, Stap J, Haveman J, van_Bree C (2006). Clonogenic assay of cells in vitro. Nat Protoc.

[88] Vermes I, Haanen C, Steffens-Nakken H, Reutelingsperger C (1995). A novel assay for apoptosis. Flow cytometric detection of phosphatidylserine expression on early apoptotic cells using fluorescein labelled Annexin V. J Immunol Methods.

[89] Euhus DM, Hudd C, LaRegina MC, Johnson FE (1986). Tumor measurement in the nude mouse. J Surg Oncol.

